# 2-(*o*-Tol­yloxy)benzoic acid

**DOI:** 10.1107/S1600536811017119

**Published:** 2011-05-14

**Authors:** Jia-Ying Xu, Wei-Hua Cheng, Xun Zhu, Dong-ya Gu

**Affiliations:** a College of Chemical and Biological Engineering, Yancheng Institute of Technology, Yinbing Road No.9 Yancheng, Yancheng 224051, People’s Republic of China; b Department of Chemical Engineering, Yancheng College of Textile Technology, Yancheng 224005, People’s Republic of China

## Abstract

In the crystal structure of the title compound, C_14_H_12_O_3_, mol­ecules are linked *via* inter­molecular O—H⋯O hydrogen bonds, resulting in dimer formation. The dihedral angle between the two phenyl rings is 76.2 (2)°.

## Related literature

For the synthesis, see: Glorius *et al.* (2009 ▶); For bond-length data, see: Allen *et al.* (1987 ▶).
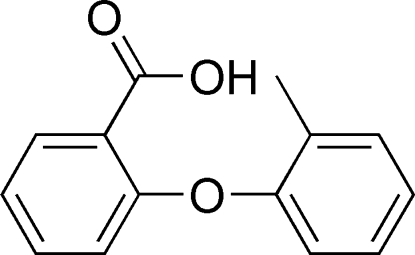


## Experimental

### 

#### Crystal data

C_14_H_12_O_3_
*M* *_r_* = 228.24
Triclinic, 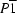

*a* = 7.0900 (14) Å
*b* = 7.4820 (15) Å
*c* = 12.680 (3) Å
α = 95.34 (3)°
β = 96.36 (3)°
γ = 115.76 (3)°
*V* = 594.5 (2) Å^3^
*Z* = 2
Mo *K*α radiation
μ = 0.09 mm^−1^
*T* = 293 K
0.30 × 0.20 × 0.10 mm

#### Data collection

Enraf–Nonius CAD-4 diffractometer
Absorption correction: ψ scan (North *et al.*, 1968 ▶) *T*_min_ = 0.974, *T*_max_ = 0.991
2382 measured reflections
2190 independent reflections
1437 reflections with *I* > 2σ(*I*)
*R*_int_ = 0.020
3 standard reflections every 200 reflections  intensity decay: 1%

#### Refinement

*R*[*F* ^2^ > 2σ(*F* ^2^)] = 0.055
*wR*(*F* ^2^) = 0.178
*S* = 1.00
2190 reflections
154 parameters
H-atom parameters constrained
Δρ_max_ = 0.23 e Å^−3^
Δρ_min_ = −0.17 e Å^−3^

### 

Data collection: *CAD-4 Software* (Enraf–Nonius, 1985 ▶); cell refinement: *CAD-4 Software*; data reduction: *XCAD4* (Harms & Wocadlo, 1995 ▶); program(s) used to solve structure: *SHELXS97* (Sheldrick, 2008 ▶); program(s) used to refine structure: *SHELXL97* (Sheldrick, 2008 ▶); molecular graphics: *SHELXTL* (Sheldrick, 2008 ▶); software used to prepare material for publication: *SHELXTL*.

## Supplementary Material

Crystal structure: contains datablocks I, global. DOI: 10.1107/S1600536811017119/vm2093sup1.cif

Structure factors: contains datablocks I. DOI: 10.1107/S1600536811017119/vm2093Isup2.hkl

Supplementary material file. DOI: 10.1107/S1600536811017119/vm2093Isup3.cml

Additional supplementary materials:  crystallographic information; 3D view; checkCIF report

## Figures and Tables

**Table 1:** Hydrogen-bond geometry (Å, °)

*D*—H⋯*A*	*D*—H	H⋯*A*	*D*⋯*A*	*D*—H⋯*A*
O2—H2*A*⋯O3^i^	0.82	1.81	2.624 (3)	172

Symmetry code: (i) 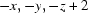.

**Fig. 1.**
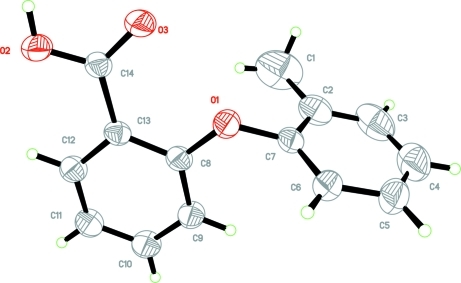
The molecular structure of (I), with the atom-numbering scheme. Displacement ellipsoids are drawn at the 50% probability level.

**Fig. 2.**
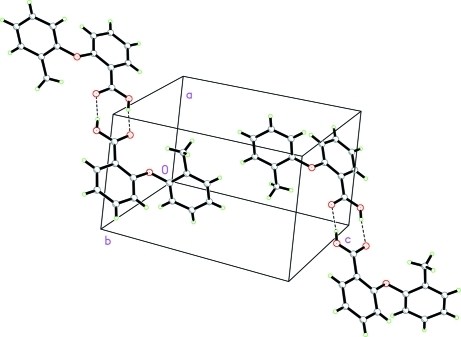
Packing diagram for (I). O—H···O hydrogen bonds are shown by dashed lines.

## supplementary crystallographic information

### Comment

The title compound, 2-(*o*-tolyloxy)benzoic acid, is an important intermediate in the synthesis of the biaryl moiety, an ubiquitous motif of polymeric materials, ligands, and biologically active compounds (Glorius *et al.*, 2009).

The molecular structure of (I) is shown in Fig. 1, and the hydrogen-bond geometry is given in Table 1. The bond lengths and angles are within normal ranges (Allen *et al.*, 1987). The dihedral angle between the two phenyl rings C8—C13 and C2—C7 is 76.2 (2) °.

The crystal packing shows dimer formation *via* O—H···O intermolecular hydrogen bonds, which seems to be very effective in the stabilization of the crystal structure. The molecules are stacked parallel to the *b* axis direction.

### Experimental

The title compound, (I) was prepared by a method reported in literature (Glorius *et al.*, 2009). The crystals were obtained by dissolving (I) (0.2 g, 0.87 mmol) in ethanol (25 ml) and evaporating the solvent slowly at room temperature for about 6 d.

### Refinement

All H atoms were positioned geometrically and constrained to ride on their parent atoms, with C—H = 0.93 Å for aromatic H and

0.96 Å for methyl H atoms , and 0.82 Å for O—H. The *U*_iso_(H) = *xU*_eq_(C), where *x* = 1.2 for aromatic H, and *x* = 1.5 for other H.

### Crystal data

**Table:**

C_14_H_12_O_3_	*Z* = 2
*M**_r_* = 228.24	*F*(000) = 240
Triclinic, *P*1	*D*_x_ = 1.275 Mg m^−^^3^
Hall symbol: -P 1	Mo *K*α radiation, λ = 0.71073 Å
*a* = 7.0900 (14) Å	Cell parameters from 25 reflections
*b* = 7.4820 (15) Å	θ = 9–14°
*c* = 12.680 (3) Å	µ = 0.09 mm^−^^1^
α = 95.34 (3)°	*T* = 293 K
β = 96.36 (3)°	Block, colorless
γ = 115.76 (3)°	0.30 × 0.20 × 0.10 mm
*V* = 594.5 (2) Å^3^	

### Data collection

**Table:**

Enraf–Nonius CAD-4 diffractometer	1437 reflections with *I* > 2σ(*I*)
Radiation source: fine-focus sealed tube	*R*_int_ = 0.020
graphite	θ_max_ = 25.4°, θ_min_ = 1.6°
ω/2θ scans	*h* = 0→8
Absorption correction: ψ scan (North *et al.*, 1968)	*k* = −9→8
*T*_min_ = 0.974, *T*_max_ = 0.991	*l* = −15→15
2382 measured reflections	3 standard reflections every 200 reflections
2190 independent reflections	intensity decay: 1%

### Refinement

**Table:**

Refinement on *F*^2^	Primary atom site location: structure-invariant direct methods
Least-squares matrix: full	Secondary atom site location: difference Fourier map
*R*[*F*^2^ > 2σ(*F*^2^)] = 0.055	Hydrogen site location: inferred from neighbouring sites
*wR*(*F*^2^) = 0.178	H-atom parameters constrained
*S* = 1.00	*w* = 1/[σ^2^(*F*_o_^2^) + (0.1*P*)^2^ + 0.094*P*] where *P* = (*F*_o_^2^ + 2*F*_c_^2^)/3
2190 reflections	(Δ/σ)_max_ < 0.001
154 parameters	Δρ_max_ = 0.23 e Å^−^^3^
0 restraints	Δρ_min_ = −0.17 e Å^−^^3^

### Special details

**Table:**

Geometry. All e.s.d.'s (except the e.s.d. in the dihedral angle between two l.s. planes) are estimated using the full covariance matrix. The cell e.s.d.'s are taken into account individually in the estimation of e.s.d.'s in distances, angles and torsion angles; correlations between e.s.d.'s in cell parameters are only used when they are defined by crystal symmetry. An approximate (isotropic) treatment of cell e.s.d.'s is used for estimating e.s.d.'s involving l.s. planes.
Refinement. Refinement of *F*^2^ against ALL reflections. The weighted *R*-factor *wR* and goodness of fit *S* are based on *F*^2^, conventional *R*-factors *R* are based on *F*, with *F* set to zero for negative *F*^2^. The threshold expression of *F*^2^ > σ(*F*^2^) is used only for calculating *R*-factors(gt) *etc*. and is not relevant to the choice of reflections for refinement. *R*-factors based on *F*^2^ are statistically about twice as large as those based on *F*, and *R*- factors based on ALL data will be even larger.

### Fractional atomic coordinates and isotropic or equivalent isotropic displacement parameters (Å^2^)

**Table:**

	*x*	*y*	*z*	*U*_iso_*/*U*_eq_	
O1	0.4820 (3)	0.1177 (2)	0.80889 (14)	0.0712 (5)	
C1	0.2152 (6)	0.0560 (7)	0.6153 (4)	0.1589 (19)	
H1A	0.1796	−0.0054	0.6782	0.238*	
H1B	0.1718	−0.0467	0.5539	0.238*	
H1C	0.1434	0.1376	0.6043	0.238*	
O2	0.0651 (3)	−0.2020 (3)	0.99961 (15)	0.0772 (6)	
H2A	−0.0042	−0.1483	1.0230	0.116*	
C2	0.4515 (5)	0.1851 (4)	0.6302 (2)	0.0833 (8)	
O3	0.1733 (3)	0.0598 (3)	0.91609 (17)	0.0833 (6)	
C3	0.5551 (8)	0.2950 (7)	0.5531 (3)	0.1137 (13)	
H3A	0.4763	0.2862	0.4874	0.136*	
C4	0.7687 (8)	0.4147 (6)	0.5719 (3)	0.1119 (13)	
H4A	0.8334	0.4873	0.5196	0.134*	
C5	0.8863 (5)	0.4283 (5)	0.6654 (3)	0.0904 (9)	
H5A	1.0323	0.5095	0.6776	0.108*	
C6	0.7915 (4)	0.3231 (4)	0.7426 (2)	0.0666 (7)	
H6A	0.8727	0.3321	0.8075	0.080*	
C7	0.5773 (4)	0.2046 (3)	0.72433 (19)	0.0570 (6)	
C8	0.4571 (3)	−0.0695 (3)	0.82224 (18)	0.0533 (6)	
C9	0.5732 (4)	−0.1542 (3)	0.7750 (2)	0.0654 (7)	
H9A	0.6639	−0.0868	0.7289	0.078*	
C10	0.5543 (5)	−0.3372 (4)	0.7963 (2)	0.0735 (7)	
H10A	0.6318	−0.3935	0.7642	0.088*	
C11	0.4216 (4)	−0.4382 (4)	0.8646 (2)	0.0722 (7)	
H11A	0.4096	−0.5619	0.8791	0.087*	
C12	0.3066 (4)	−0.3541 (3)	0.91134 (19)	0.0605 (6)	
H12A	0.2185	−0.4218	0.9583	0.073*	
C13	0.3188 (3)	−0.1708 (3)	0.89020 (17)	0.0497 (5)	
C14	0.1800 (3)	−0.0953 (3)	0.93742 (18)	0.0522 (5)	

### Atomic displacement parameters (Å^2^)

**Table:**

	*U*^11^	*U*^22^	*U*^33^	*U*^12^	*U*^13^	*U*^23^
O1	0.0866 (12)	0.0514 (9)	0.0985 (13)	0.0410 (9)	0.0489 (10)	0.0272 (9)
C1	0.102 (3)	0.141 (4)	0.193 (5)	0.043 (3)	−0.048 (3)	−0.014 (3)
O2	0.0842 (12)	0.0730 (11)	0.1051 (14)	0.0510 (10)	0.0480 (11)	0.0389 (10)
C2	0.092 (2)	0.0775 (18)	0.0812 (19)	0.0452 (16)	−0.0036 (16)	0.0006 (15)
O3	0.0924 (14)	0.0684 (11)	0.1259 (16)	0.0542 (10)	0.0591 (12)	0.0458 (11)
C3	0.174 (4)	0.131 (3)	0.064 (2)	0.099 (3)	−0.001 (2)	0.015 (2)
C4	0.161 (4)	0.127 (3)	0.093 (3)	0.089 (3)	0.065 (3)	0.053 (2)
C5	0.095 (2)	0.083 (2)	0.110 (2)	0.0428 (17)	0.0529 (19)	0.0361 (18)
C6	0.0678 (16)	0.0677 (15)	0.0724 (16)	0.0338 (13)	0.0220 (13)	0.0191 (12)
C7	0.0694 (15)	0.0468 (11)	0.0687 (15)	0.0350 (11)	0.0240 (12)	0.0141 (10)
C8	0.0582 (13)	0.0420 (11)	0.0655 (14)	0.0267 (10)	0.0141 (11)	0.0091 (10)
C9	0.0781 (16)	0.0549 (13)	0.0775 (16)	0.0382 (12)	0.0308 (13)	0.0132 (12)
C10	0.0914 (19)	0.0580 (14)	0.0921 (19)	0.0486 (14)	0.0324 (15)	0.0121 (13)
C11	0.0890 (18)	0.0517 (13)	0.0904 (19)	0.0415 (13)	0.0252 (15)	0.0169 (13)
C12	0.0660 (14)	0.0481 (12)	0.0724 (15)	0.0286 (11)	0.0168 (12)	0.0120 (11)
C13	0.0482 (11)	0.0419 (11)	0.0590 (13)	0.0213 (9)	0.0064 (10)	0.0052 (9)
C14	0.0496 (12)	0.0450 (11)	0.0649 (14)	0.0220 (9)	0.0132 (10)	0.0141 (10)

### Geometric parameters (Å, °)

**Table:**

O1—C8	1.363 (2)	C5—C6	1.363 (4)
O1—C7	1.392 (3)	C5—H5A	0.9300
C1—C2	1.505 (5)	C6—C7	1.364 (3)
C1—H1A	0.9600	C6—H6A	0.9300
C1—H1B	0.9600	C8—C9	1.390 (3)
C1—H1C	0.9600	C8—C13	1.393 (3)
O2—C14	1.272 (2)	C9—C10	1.372 (3)
O2—H2A	0.8200	C9—H9A	0.9300
C2—C7	1.364 (4)	C10—C11	1.375 (4)
C2—C3	1.398 (5)	C10—H10A	0.9300
O3—C14	1.235 (2)	C11—C12	1.376 (3)
C3—C4	1.361 (5)	C11—H11A	0.9300
C3—H3A	0.9300	C12—C13	1.390 (3)
C4—C5	1.340 (5)	C12—H12A	0.9300
C4—H4A	0.9300	C13—C14	1.482 (3)
			
C8—O1—C7	119.48 (17)	C2—C7—O1	118.9 (2)
C2—C1—H1A	109.5	C6—C7—O1	118.4 (2)
C2—C1—H1B	109.5	O1—C8—C9	121.8 (2)
H1A—C1—H1B	109.5	O1—C8—C13	117.77 (18)
C2—C1—H1C	109.5	C9—C8—C13	120.32 (19)
H1A—C1—H1C	109.5	C10—C9—C8	120.0 (2)
H1B—C1—H1C	109.5	C10—C9—H9A	120.0
C14—O2—H2A	109.5	C8—C9—H9A	120.0
C7—C2—C3	116.0 (3)	C9—C10—C11	120.6 (2)
C7—C2—C1	120.1 (3)	C9—C10—H10A	119.7
C3—C2—C1	123.9 (3)	C11—C10—H10A	119.7
C4—C3—C2	121.5 (3)	C10—C11—C12	119.3 (2)
C4—C3—H3A	119.2	C10—C11—H11A	120.4
C2—C3—H3A	119.2	C12—C11—H11A	120.4
C5—C4—C3	120.4 (3)	C11—C12—C13	121.7 (2)
C5—C4—H4A	119.8	C11—C12—H12A	119.1
C3—C4—H4A	119.8	C13—C12—H12A	119.1
C4—C5—C6	119.9 (3)	C12—C13—C8	118.0 (2)
C4—C5—H5A	120.1	C12—C13—C14	118.97 (19)
C6—C5—H5A	120.1	C8—C13—C14	123.02 (18)
C5—C6—C7	119.8 (3)	O3—C14—O2	122.0 (2)
C5—C6—H6A	120.1	O3—C14—C13	122.05 (19)
C7—C6—H6A	120.1	O2—C14—C13	115.94 (18)
C2—C7—C6	122.3 (2)		
			
C7—C2—C3—C4	−0.3 (5)	O1—C8—C9—C10	175.6 (2)
C1—C2—C3—C4	177.9 (4)	C13—C8—C9—C10	−0.9 (4)
C2—C3—C4—C5	0.7 (6)	C8—C9—C10—C11	−0.3 (4)
C3—C4—C5—C6	−0.5 (5)	C9—C10—C11—C12	0.3 (4)
C4—C5—C6—C7	−0.1 (4)	C10—C11—C12—C13	0.9 (4)
C3—C2—C7—C6	−0.3 (4)	C11—C12—C13—C8	−2.0 (4)
C1—C2—C7—C6	−178.6 (3)	C11—C12—C13—C14	175.9 (2)
C3—C2—C7—O1	172.8 (2)	O1—C8—C13—C12	−174.58 (19)
C1—C2—C7—O1	−5.5 (4)	C9—C8—C13—C12	2.0 (3)
C5—C6—C7—C2	0.5 (4)	O1—C8—C13—C14	7.6 (3)
C5—C6—C7—O1	−172.7 (2)	C9—C8—C13—C14	−175.9 (2)
C8—O1—C7—C2	94.5 (3)	C12—C13—C14—O3	−175.2 (2)
C8—O1—C7—C6	−92.1 (3)	C8—C13—C14—O3	2.6 (4)
C7—O1—C8—C9	18.4 (3)	C12—C13—C14—O2	3.5 (3)
C7—O1—C8—C13	−165.1 (2)	C8—C13—C14—O2	−178.7 (2)

### Hydrogen-bond geometry (Å, °)

**Table:**

*D*—H···*A*	*D*—H	H···*A*	*D*···*A*	*D*—H···*A*
O2—H2A···O3^i^	0.82	1.81	2.624 (3)	172

Symmetry codes: (i) −*x*, −*y*, −*z*+2.
